# Buffalo Physicians in the Government Service

**Published:** 1861-10

**Authors:** 


### BUFFALO PHYSICIANS IN THE GOVERNMENT SERVICE.

In the August number, we gave a list of Buffalo Physicians in the service of their country. As there have been promotions and alterations since then we revise the list:

*United States Army.*

Charles K. Winne, M. D. Assistant Surgeon, under Gen. Rosecrans.

*New York State Volunteers.*

Charles H. Wilcox, M. D., Surgeon 21st Regiment.

Lucien Damainville, M. D., Surgeon 31st Regiment.

Joseph A. Peters, M. D., Assistant Surgeon 21st Regiment.

A. J. Steele, M. D., Assistant Surgeon 26th Regiment.

Elias L. Bissell, M. D., Assistant Surgeon Ellsworth Regiment.

*Michigan Volunteers.*

Wm. H. Butler, M. D. Assistant Surgeon.

*New Mexico Volunteers.*

Sylvester Rankin, M. D., Assistant Surgeon, 2d Regiment.

*United States Navy.*

Newton L. Bates, M. D., Assistant Surgeon, U. S. Naval Hospital Brooklyn.

William Howell, M. D., Assistant Surgeon, U. S. Sloop "Richmond."

Samuel D. Flagg, Jr., M. D. Assistant Surgeon, U. S. Gunboat "Connecticut."

*U. S. Volunteer Navy.*

Ira C. Whitehead, M. D., Assistant Surgeon. At Sea.
Geo. L. Sweet, M. D., Assistant Surgeon. Waiting orders.

It affords us great pleasure to see the names of so many of our professional brethren enrolled in the list of those who enter the *struggle for right*, to mitigate the horrors of war, and afford aid and comfort to our brave soldiers. It speaks well for the profession in our city to say, that all who have thus far applied to enter the service, have passed with credit the trying examination; and we doubt if, in proportion to its size, any other city has sent forth as many medical men to the war as ours. It is also a source of gratification to learn, that all of the graduates of the Buffalo Medical College, who are known to have applied, have passed successfully the searching investigation into their professional acquirements; and last week, when Dr. W. B. Mann' of Brockport, (a graduate of last spring,) passed the Navy Board. its president paid the Buffalo school a very high compliment, saying that it was "the only school from which candidates have not been rejected during the recent examinations for the government service." In this respect, it stands *first.* It should be a subject for congratulation, that we have such an institution in our midst. In these investigations, when graduates from all schools assemble, and severe and impartial examinations are held; it is easy to determine where the system of education is *thorough*, and young medical men are carefully disciplined, and rendered *competent* to practice their noble art. The classical advantages of this school are equal to any other; its instructors are among the first in their profession; the institution is flourishing and prosperous, steadily gaining in patronage from year to year, and at the same time elevating the standard of professional acquirements. In its results, we are happy to see that it favorably compares with any other—it certainly is second to none. "B."

